# Monthly Record of Deaths in the City of Buffalo

**Published:** 1858-05

**Authors:** H. D. Garvin

**Affiliations:** Health Physician


### ART. III.—*Report of Mortality in Buffalo for the month of March*, 1858. By H. D. Garvin, M. D., Health Physician.

| DISEASES. | No. | Males. | Females. | No Sex given. |
|---|---|---|---|---|
| Accidental, | 5 | 5 | | |
| Asthma, | 1 | 1 | | |
| Born Sickly, | 2 | 1 | 1 | |
| Bronchitis, | 3 | | 3 | |
| Croup, | 2 | 1 | 1 | |
| Congestion of Lungs, | 3 | 2 | 1 | |
| " " Brain, | 1 | | 1 | |
| Convulsions, | 16 | 9 | 7 | |
| Diarrhœa, | 2 | 1 | | 1 |
| Dysentery, | 2 | | 2 | |
| Dropsy, | 1 | | 1 | |
| Eclampsia, | 1 | | 1 | |
| Enteritis, | 2 | 1 | 1 | |
| Exhaustion, | 1 | | 1 | |
| Fever, Typhoid, | 3 | 1 | 2 | |
| " Scarlet, | 4 | 3 | 1 | |
| Gastritis, | 3 | 2 | 1 | |
| Heart Disease, | 3 | 2 | 1 | |
| Hydrocephalus, | 8 | 3 | 3 | 2 |
| Influenza, | 2 | 1 | 1 | |
| Intemperance, | 5 | 3 | 2 | |
| Insanity and Epilepsy, | 1 | 1 | | |
| Marasmus, | 3 | 1 | 1 | 1 |
| Measles, | 2 | 1 | 1 | |
| Old Age, | 6 | 3 | 3 | |
| Paralysis, | 1 | 1 | | |
| Phthisis Pulmonalis, | 17 | 9 | 8 | |
| Pneumonia, | 9 | 4 | 3 | 2 |
| " Typhoides, | 1 | 1 | | |
| Premature Birth, | 2 | 1 | | 1 |
| Puerperal Fever, | 2 | | 2 | |
| " Convulsions, | 1 | | 1 | |
| Pulmonary Apoplexy, | 1 | 1 | | |
| Rheumatism, | 1 | 1 | | |
| Scrophula, | 3 | 2 | 1 | |

REGISTER OF MORTALITY—CONTINUED.

| DISEASES. | No. | Males. | Females. | No Sex given. |
|---|---|---|---|---|
| Spinal Disease, | 1 | | 1 | |
| Still-born, | 8 | 5 | 2 | 1 |
| Syphilis Testiary, | 1 | 1 | | |
| Uterine Cancer, | 1 | | 1 | |
| Total, | 134 | 69 | 55 | 8 |

SEXES.

| | |
|---|---|
| Males, | 69 |
| Females, | 55 |
| Sex not given, | 8 |
| Total, | 134 |

AGES.

| | | | |
|---|---|---|---|
| Still-born, | 8 | Between 20 years and 30 years, | 13 |
| 1 day, | 4 | " 30 " " 40 " | 13 |
| 1 day and 30 days, | 3 | " 40 " " 50 " | 7 |
| Between 1 month and 6 months, | 10 | " 50 " " 60 " | 10 |
| " 6 months and 12 " | 12 | " 60 " " 70 " | 6 |
| " 1 year " 3 years, | 13 | " 70 " " 80 " | 7 |
| " 3 " " 5 " | 5 | " 80 " " 90 " | 1 |
| " 5 " " 10 " | 9 | " 90 " " 100 " | 0 |
| " 10 " " 20 " | 3 | " 100 " | 0 |
| | 67 | | 57 |
| Ages not given, | 10 | | 134 |
| Total, | 134 | | |

NATIVITIES.

| | | | |
|---|---|---|---|
| American, | 79 | Prussian, | 0 |
| German, | 24 | Swiss, | 0 |
| Irish, | 18 | French, | 1 |
| English, | 5 | Scotch, | 2 |
| Canadian, | 2 | Bohemia, | 0 |
| Holland, | 1 | Country not given, | 2 |
| Total, | | | 134 |

